# American Medical Association

**Published:** 1872-05

**Authors:** 


*American Medical Association. Twenty-third Annual Congress.* Held in Philadelphia, May 7—10, 1872.

[For the following abstract we are indebted to the Special Correspondence of The Clinic, of May 18th.—Ed.]

TUESDAY.

At eleven o'clock this morning there was opened the first session of the meetings of the American Medical Association. A very large number of gentlemen are in attendance, as well as many of the softer sex, all come to take part in or listen to the proceedings of the association, as these should gradually unfold themselves. The number present exceeds that of any previous convention ever held. During the morning the delegates assembled in Horticultural Hall, and there for a considerable time occupied themselves in the courteous interchange of friendly greetings with brother professionals, with whom they had not associated for many years.

The association is composed of delegates from local societies over the whole United States, and from colleges, hospitals, lunatic asylums, and other permanently organized medical institutions of good standing in the United States, from the army and navy, and from the American Medical Society of Paris. Prof. Wm. H. Gobrecht is registered as the delegate from the Medical College of Ohio, Drs. Comegys and Carson from the hospital, and Drs. Walker, Nardyz and others from the Academy of Medicine. The large number of delegates present occupy about two-thirds of the floor of the hall, which has been railed off for their use. The remainder of the floor and gallery was at the disposal of spectators, among whom were a large number of ladies. Every arrangement was made for the convenience of the body in transacting its business.

The attendance is nearly one thousand in number, of men of more than ordinary talent and appearance of intelligence.

Shortly after eleven o'clock the association was called to order by the President, Dr. D. W. Yandell, of Louisville, Kentucky.

The proceedings were opened with an appropriate and impressive prayer by the Right Rev. Wm. Bacon Stevens, M.D., D.D., D.C.L., Protestant Episcopal Bishop of Pennsylvania.

The address of welcome on behalf of the medical profession of Philadelphia, was delivered by Professor R. E. Rogers, M.D., chairman of the Committee of Reception.

The greeting on behalf of the Committee of Arrangements was delivered by Edward Hartshorne, M.D., of that committee, who then recited the programme of entertainments arranged for the strangers during their stay in the city.

Dr. Wm. B. Atkinson, of Philadelphia, permanent Secretary, read a list of the delegates whose credentials have been approved.

President Yandell read the annual address, which was a very long and erudite paper, pleasantly relieved with dashes of anecdote and humor.

A vote of thanks was passed for the address of the President.

The report of the Committee on Credentials was presented as far as read.

Drs. Morris J. Ash, U.S.A., M. W. Junker, of Bellaire, O., and David D. Mahan, of Mifflin county, Pa., were elected members by invitation.

Dr. H. F. Askew, of Delaware, offered a resolution that all questions of a personal character, including complaints and protests, and all questions on credentials, be referred at once to the Committee on Ethics, and without discussion.

Dr. Mussey, of Ohio, asked that the motion be divided, referring questions of personal character only to the Committee on Ethics.

Dr. Askew declined to accept the amendment for a division of the question.

Dr. Mussey asked if the question did not concern the rights of societies to be admitted in convention, and if so, he denied the right of the committee to decide upon such questions. He proposed, also, referring the second branch of the subject to the convention.

The Chair said that such a course had never been pursued. Hitherto the Committee on Ethics reported to the convention on both subjects, their action being open to the approval of the convention.

Dr. Hartshorne said that the course of the Committee of Arrangements was plain and defined by law, and the original resolution was framed in accordance therewith.

The previous question being ordered by the house, Dr. Askew's motion was put and carried without a dissenting voice.

Various reports were next announced on the subject of papers to be read at the sections held in the afternoon.

The following gentlemen were appointed the Committee on Ethics:

Dr. Askew, of Delaware; N. S. Davis, of Illinois; Calvin Slavy, of Maine; I. K. Bastette, of Delaware; and S. D. Gross, of Philadelphia. The session then adjourned.

In the afternoon, the various sections of Practice and Obstetrics, Surgery, Anatomy and other medical and scientific branches, met in different portions of the hall as designated on the delegates' tickets, for the purpose of hearing reports of Special Committees on Climatology, Epidemics, Surgical Operations, Ophthalmology, etc.

In the evening, a reception was given at Horticultural Hall, by the Biological Society. In the foyer were arranged two long tables, on which were placed over one hundred powerful microscopes, containing specimens of pathology, anatomy, microscopic photography, etc.

Upon the platform at the end of the room were arranged five spectroscopes, in charge of Professor B. H. Rand, of the Jefferson Medical College, who exhibited the spectra of the different mineral and other substances, and of the electrical light.

During the evening a promenade concert was given in the main hall by Hassler's Orchestra. The visitors were greatly interested in the display, and pleased with the entertainment. Among the specimens examined under the microscope, were fleas, beetles, fang of a rattlesnake; human, insect, and animal blood; Egyptian mummy hair, the human nerve trunk, sections of the eye, lungs, heart, kidneys, cancer of the mammary glands; photographs of Nobert's test plates, ninth and nineteenth bands, from the Surgeon General's office at Washington, etc.

The following is the Committee of Arrangements appointed by the Association:

E. Hartshorne, M.D., Chairman; Richard H. Townsend, M.D., John H. Rackard, M.D., William Pepper, M.D., F. F. Maury, M.D., James Tyson, M.D., S. W. Gross, M.D.; D. Murray Cheston, M.D., Secretary.

WEDNESDAY.

The delegates to the American Medical Association assembled in Horticultural Hall at 10 o'clock this morning. The attendance was even larger than on the day previous.

Shortly after 10 o'clock the association was called to order by Dr. Thomas M. Logan, of California, first Vice President, who stated that a recess of fifteen minutes would be taken for the purpose of organizing State delegations and electing members of a general committee of one from each delegation to nominate officers for the ensuing year, and name the city for the next annual meeting.

At the close of the recess the association proceeded to Dr. Wylie's church, Broad street, below Spruce, where the session was resumed. This move was rendered necessary owing to the difficulty of hearing in Horticultural Hall. Nearly seven hundred delegates took seats in the church at 11 o'clock, and the work of the convention proceeded with much less confusion than was reasonably anticipated as the result of the sudden change of base.

At the church, Dr. Yandell, President, of Louisville, took the chair, and the Secretary read the list of members selected as the Nominating Committee, when they retired for consultation.

Dr. W. H. Pancoast announced, through the Committee of Arrangements, that the ladies were also invited to be present at his reception in the evening. It was subsequently announced, that Dr. Hugh L. Hodge had also made arrangements to provide for the ladies accompanying the delegates to his reception.

Dr. R. E. Rogers offered a resolution inviting Dr. J. Edgar Chancellor, of the University of Virginia, to a seat on the floor of the house, which was adopted.

Dr. R. Bronson, of Massachusetts, offered a resolution providing that the Committee on Ethics, to consist of seven members, shall be chosen hereafter by the Committee on Nominations, instead of by the President, as heretofore.

The President thought this was virtually an amendment to the by-laws, and should lie over under the rules.

Dr. Bronson replied, that the resolution might be passed at this session, to take effect at the next session of the association. This idea was entertained by the Chair, and after an explanation by Dr. Bronson, that his resolution was offered for the sole purpose of securing harmony, a vote was taken, resulting in the defeat of the resolution by a vote of 163 yeas and 187 nays.

Mr. Van Bibber, of Baltimore, moved that a special committee be appointed to investigate the merits of a certain nameless substitute for quinine.

The Chair stated, that under the rules the matter must be referred to the section on Materia Medica.

Dr. Davis, of Chicago, read a preamble and resolution indorsing the efforts of the Massachusetts State Society in crowding out quacks, charlatans, and pretenders from the profession, which subject was referred to the Committee on Ethics.

The Secretary asked that Dr. Edward Seguin, of New York, be permitted to exhibit his method of examining patients with a thermometer.

Dr. Davis opposed this violation of the rules as establishing a troublesome precedent.

The President remarked that he was accused the day before of being discourteous, and he was now disposed to exercise all the courtesy he could.

Dr. Seguin was told to present his method to the proper section.

The regular business of the association was then resumed.

Dr. Francis Gurney Smith of this city, chairman of the Committee on Publication, presented his report. It set forth that 750 copies of the Transactions of the Society had been published, at a cost of $1,549.39. Of these, 475 volumes were distributed to members, including 23 to various medical journals, and 88 copies are still due to members. The work was completed and issued early in November. The report concluded by reminding the members of a resolution passed in 1870, that all members who failed to comply with the rules of the association within one year forfeited their right to a copy.

Dr. Caspar Wistar, of Philadelphia, Treasurer of the association, reported that the association last year exercised a praiseworthy discrimination in the selection of the material furnished for publication, and consequently the volume of transactions was smaller, more compact, cheaper and more desirable to the profession than heretofore. The edition was published at a price which leaves a balance on hand for the use of the association when it may hereafter become forgetful of its prudence, and refer a great mass of manuscript for publication.

The treasurer counseled care in the adoption of prize essays, for out of this has arisen considerable expense. The treasury is depleted annually to the extent of $200 which it can ill afford. The account current shows a balance in hand of $1,005, being about $300 more than was in hand last year. The expenditures during the year were $3,680. The Treasurer asks the association to bear in mind that there are no discretionary powers vested in the Committee on Publication. They must publish everything which is referred to them by the sections.

Referred to the publication committee.

Dr. J. S. Weatherly, of Alabama, chairman of the Committee on Medical Education, read the report of that committee. It urges the immediate suppression of the cheap medical colleges, through a petition to the State Legislatures asking for the withdrawal of all charters of institutions of a doubtful character. The committee recommended the adoption of a badge or medal, to be given to all physicians in the country who are deemed eligible for membership in the American Medical Association.

It is also recommended that the association shall establish a high standard for medical education and members, to be arranged by a congress of professors and practicing physicians and surgeons, all of whom are members of the American Medical Association.

Dr. Francis G. Smith, of Philadelphia. "In the absence of the chairman of the Committee on Prize Essays, who is detained at home by a sad bereavement in his family—the death of his son Louis—and who has commissioned me, as the second member of his committee, to act in his place, I will present the report to the association."

Four essays were submitted to the committee; of these, one was withdrawn by its author, and the remaining three were carefully examined by the committee. Two of them did not fulfill the conditions which are to determine the disposition of the prize. The third one did—that is, it presented the condition of original research. The title of that essay is, "What Physiological Value has Phosphorus as an Organismal Element," and it bears a Latin motto—"Ne tentis, aut perfice." The name of the author is Samuel R. Percy, of New York City.

Dr. T. Parvin, of Indiana, Chairman of the Committee on Medical Literature, reported that he had addressed a letter to each of his four associates—Drs. J. P. Whitney, of California; G. Mendenhall, of Ohio; H. Carpenter, of Oregon, and L. P. C. Garvin, of Rhode Island. He received replies from the two last named only. Dr. Carpenter said he had nothing to communicate. Dr. Garvin sent a long letter which was read, and which contained observations in reference to the national literature and suggestions for its improvement. The writer asserted that we have an American medical literature of which every one should be proud. This very city has produced works which would make quite a library of themselves, and without which no medical library, however vast and various its volumes, would be complete. The names of four of the living authors of Philadelphia who specially deserve mention are George B. Wood, Hugh L. Hodge, Isaac Hayes, and Samuel D. Gross. Our literature, he says, is practical in character. Although we may boast of the grace and beauty with which Dr. Chapman clothed his thoughts; of the flashing declarations of the late Dr. Meigs; of the calm dignity and ornate periods of Dr. Wood, yet generally, our American authors give less heed to language than to ideas. If an author wishes to catch the ear of an American physician, he must have something useful to say, and must say it quickly.

The committee favored the idea of offering a triennial prize of $600 for the best essay, instead of the present plan of giving $200 to be divided between two each year. They suggest that the chairman of each section deliver an address to his section, as likely to relieve some of the irksomeness of listening to dry essays. A large portion of this report was devoted to advocating the publication of a national monthly journal, under the auspices of the American Medical Association.

Referred to the publication committee.

Dr. J. D. Jackson, of Kentucky, chairman of the Committee on American Necrology, submitted his report, which was referred to the Committee on Publication without being read.

The Secretary announced that Dr. J. G. Richardson, of Philadelphia, would read, before one of the sections, a paper on the structure of the white blood corpuscles.

Also, that Dr. Hartshorne would read one on the physiology of the vaso motor nerves.

Also, that Dr. Pancoast would present to one of the sections, through Dr. S. Bonaparte Merkel, the case of George Thomas. This is a colored man, a native of England, now a resident of Frederick City, Md., thirty-six years old, who makes a living by exhibiting his ability to roll his abdomen with an undulatory motion, like a huge ball, around the umbilicus, and also to move his heart from side to side.

Also, that Dr. J. S. Packard, of Philadelphia, would exhibit to the surgical section a suspension apparatus for fracture of the leg.

Also, that Dr. Hunt had sent in his report on the climatology of New Jersey.

Referred.

Also, that Dr. George M. Beard would read a paper on the recent researches in electro-therapeutics, with a demonstration of some of the methods of application.

Also, that Dr. John P. Garrish would read a paper on "The use of instruments in obstetrics."

Also, that Dr. W. H. Mussey would read a volunteer paper on "A case of vesical calculus."

Also, that Dr. Harrison Allen would read a paper on "The soft palate in health and disease."

Also, that Dr. Wm. F. Peck, of Iowa, would read his report of an operation on the hip joint, with details of the case.

Dr. Wm. R. Findley, of Pennsylvania, presented a resolution which declared that as the association, at its meeting in 1869, unanimously adopted a resolution classing all who practiced medicine on the "contract system" with "irregular practitioners,"

and as this action has excited considerable controversy in many of the county societies, the members of which have misunderstood the meaning of that resolution, it is therefore important that the Committee on Ethics decide whether such physicians are really "irregular."

Referred to the Committee on Ethics.

Dr. George Sutton, of Indiana, read a report on "Comparative Pathology," in which it is urged that a national medical library should be established.

Dr. Woodward, of the United States Army, observed that such a library is now in existence in the Surgeon-General's office at Washington, and contains eighteen thousand volumes.

Dr. Pollock, of Pennsylvania, offered a resolution creating a special committee to consider the propriety of adopting the suggestions made in the report of the Committee on Medical Education, which was postponed for the present.

A long discussion ensued upon the matter of allowing the Committee on Publication to publish papers in the annual report at their discretion. The subject was finally postponed indefinitely.

Adjourned.

In the afternoon the various sections and Committee on Ethics held meetings, at which a vast number of subjects were discussed, and among them the question of admitting the representative of the Howard University (colored) and several colored physicians from Washington.

In the evening a lecture was delivered at the University of Pennsylvania by Dr. Noyes, of New York, on "The relation of diseases of the inner structure of the eye to other affections of the body." There was a large audience present, composed of the delegates, their families and friends. The lecturer showed the analogy between the diseases of the eye and those of other portions of the body, illustrating his remarks by means of ophthalmoscopic pictures projected upon screens by means of the magic lantern.

At the close of this address the audience passed into the chemical lecture room, where Prof. R. E. Rogers delivered a short lecture on the two subjects of Spectroscopy and Electricity. He first explained the action of the spectroscope in analyzing the light of the sun and other heavenly bodies, and also that produced by the combustion of minerals and gases, and in illustration he projected upon a screen the spectra of several of the substances alluded to.

During the evening, receptions were given to the delegates and their ladies at the residences of Dr. William Pancoast and Dr. H. L. Hodge.

THURSDAY.

The association re-assembled this morning at the appointed hour in Dr. Wylie's church; President Yandell in the chair.

A large number of physicians were, on motion, invited to seats on the floor of the convention.

After some preliminary business, Dr. Stein, of New York, offered a resolution that a committee be appointed to examine and report upon the transmission of diseases from animals to man, and to report what measures may best be adopted to prevent and arrest disease in animals.

Agreed to.

A preamble and resolutions adopted by the College of Physicians of this city, the preamble reciting the frequency of cases of accidental poisoning by druggists, and the resolution recommending that all bottles containing poison should not only be labeled poison, but should be roughed on one side so as to indicate their poisoning contents to the sense of touch, and also be labeled with the most ready and efficient antidote, was, upon motion, adopted by the convention.

A resolution offered by Dr. Horner, of Virginia, that the members of the association should discourage the use of alcoholic stimulants in their remedies, was adopted. There was one "No," the enunciation of which produced considerable merriment.

Dr. Walker, of Brooklyn, presented a resolution that so much of the report of the Committee on Literature as relates to the establishment of a national medical journal be referred to a committee, to report at the present session.

Agreed to.

Dr. Francis Gurney Smith, of Philadelphia, chairman of the Committee on Medical Nomenclature, made a report with a resolution attached creating a special committee to prepare the classification and lists of names of diseases for publication and adoption by the association.

Dr. Woodward, of the United States army, read a minority report, with a resolution appended, "that the classification and nomenclature proposed by the majority of the committee be printed in pamphlet form, and spread broadcast throughout the profession, and that the report be postponed for one year."

After a protracted discussion, Dr. Woodward's resolution w adopted.

Dr. Keller, of Kentucky, then presented the following:

*Resolved*, That so much of the report of the Committee on Education as refers to the establishment of a medical congress, consisting of an equal number of representatives from each State, is of immediate importance, and, in order to further the views thus expressed, that a committee be appointed at once to take the preliminary steps necessary to organization, with instructions to report before the adjournment of this convention.

Before acting upon this, Dr. William O. Baldwin, of Alabama, asked leave to make a partial report from the Committee on Nominations. This was granted, and he announced that the committee had agreed upon the following officers to serve during the ensuing year:

President—Dr. Thomas L. Logan, Cal.
First Vice-President—Dr. Catlin, Conn.
Second Vice-President—Dr. McPheeters, Mo.
Third Vice-President—Dr. Pollock, Pa.
Fourth Vice-President—Dr. Briggs, Tenn.
Treasurer—Dr. Caspar Wistar, Pa.
Librarian—Dr. Wm. Lee, D. C.
Assistant Secretary—Dr. M. A. Fallon, Mo.

Next place of meeting—St. Louis.

The report was accepted.

Dr. Baldwin requested further time for his Committee to complete their report.

Dr. Keller's resolution was then voted upon and not agreed to.

Dr. Palmer, in behalf of the delegation from Missouri, thanked the association for selecting St. Louis for the next place of meeting.

Dr. Palmer then presented a proposition for the establishment of a national medical academy, for the purpose of elevating the standard of medical attainments and the moral worth of its members.

The association refused to adopt this scheme.

Dr. T. M. Logan, of California, chairman of the committee on A National Health Council, made a long report. The conclusions of this committee can be gathered from the following extract from the report:

"Diffuse the discovery of the means of protection against these and many other diseases which have been perfected under the vigilant outlook and investigation of combined chemical and microscopic detectives; extend what has been successfully applied in circumscribed communities to states and to nations; let facilities for concerted action be established internationally through the instrumentality of governments, and the people will no more be decimated by those pandemic waves which have so often swept with cumulative impetuosity over the face of the earth. Utopian as the idea may at first sight appear of stamping out the great anti-sanitary evils which beget disease, still it would be taking a very limited view of the power of the human mind, and argue a strange obliquity of vision as to the lessons its triumphs in other fields are every day teaching us, to doubt our ultimate ability to do so. 'That man, who is rapidly subduing all the most Titanic forces of the universe to his commonest uses, should always remain at the mercy of these ignoble things, is an antithesis too extreme to be permanent.'

"The government of the United States has already done something in the direction toward which these suggestions tend, by the establishment of a bureau connected with the war department, which makes constant synopses of the weather, storm currents and other meteorological phenomena occurring in some of the most prominent parts of the Union. Let the operations of the 'signal service' be so extended as to reach the remotest expansions of the republic; and while there shall be sent from the capital of every State and territory full telegrams of the daily travail of nature in all her parts to the federal head, let the respondent wire report back simultaneously everything of scientific interest to the physician as well as to the physicist."

The committee asked to be continued, and to be constituted a special section on state medicine and public hygiene, to which all subjects cognate thereto may be referred; also that they be empowered to take such action, in connection with the authorities at Washington, as in their judgment may be deemed expedient in carrying out the objects of the resolutions.

D. J. C. Tucker, of California, offered a resolution appealing to Congress to establish a national sanitary bureau, in charge of competent medical men, to be appointed by the United States treasurer, from physicians nominated by this association.

The bureau was to have charge of all governmental hospitals, and was to secure the proper supply of bovine virus, besides performing many other national duties.

The plan of Dr. Tucker was very elaborate, and contained an act bearing upon this subject to be presented to Congress.

Dr. Gallagher moved that the report of Dr. Tucker be laid upon the table.

Agreed to.

Dr. Tyler, of Washington, D. C., insisted upon the members exercising great care in submitting questions to Congress, as all such subjects were likely to be governed by a ring for their personal advantage.

That portion of the report of the committee which referred to taking action in connection with the authorities in Washington was stricken out, and the report was then agreed to.

The Committee on Nominations presented a final report as follows:

Committee of Arrangements.—Dr. J. B. Johnston, Dr. J. T. Hodges, Dr. J. S. Moore, Dr. Robinson, Dr. Kennard, Dr. Teste, Dr. Brokay, Dr. J. M. Scott, all of St. Louis.

Committee on Publication.—Dr. Atkinson, chairman, Pa.; Dr. D. Murray, Chester, Pa.; Dr. Wm. Lee, District of Columbia; Dr. Caspar Wistar, Pa.; Dr. H. F. Askew, Delaware; Dr. A. Meigs, Pa.

Committee on Prize Essays.—Dr. Moore, chairman, Missouri; Dr. Gregory, Missouri; Dr. Davis, Illinois; Dr. Parvin, Indiana; Dr. Mendenhall, Ohio.

Committee on Medical Education.—Dr. Carson, chairman, Ohio; Dr. Tojan, Ga.; Dr. Howard, Md.; Dr. Steel, Col.; Dr. Vanderpool, N. Y.; Dr. Johnson, District of Columbia; Dr. Stout, Ga.; Dr. Welsh, Texas; Dr. Scott, Ark.; Dr. Bailey, N. Y.; Dr. Jones, Ala.; Dr. McRuor, Me.; Dr. Tally, S. C.; Dr. Blaine, N. J.; Dr. Shattuck, Mo.; Dr. Jasques, Va.

Committee on Medical Literature.—Dr. Flint, chairman, N. Y.; Dr. Yandell, Sr., Ky.; Dr. Henderson, Ala.; Dr. Thrall, Ga.; Dr. Leary, Me.

Committee on Medical Necrology.—Dr. Jackson, chairman, Ky.; Dr. Parsons, R. I.; Dr. Hildreth, W. Va.; Dr. Johnson, D. C.; Dr. Simmons, Cal.; Dr. Wariner, Oregon; Dr. Stevens, Ohio; Dr. Agnew, Pa., and others.

Officers of Sections:

Chemistry and Materia Medica.—Dr. R. E. Rogers, chairman, Philadelphia; Dr. Ephraim Cutter, Secretary, Boston.

Practice of Medicine and Obstetrics.—Dr. D. A. O'Donnell, chairman, Baltimore; Dr. Benjamin F. Dawson, secretary, New York.

Surgery and Anatomy.—Dr. Warner, chairman, Baltimore; Dr. W. Peck, secretary, Iowa.

Climatology and Epidemics.—Dr. Geo. Sutton, chairman, Ind.; Dr. Elisha Harris, secretary, New York.

Medical Jurisprudence, Hygiene and Physiology.—Dr. R. C. Busey, chairman, Washington; Dr. H. B. Arnold, secretary, Baltimore.

Psychology.—Dr. Isaac Ray, chairman, Philadelphia; Dr. John Curwin, Secretary, Harrisburg.

The report was accepted.

A preamble and resolutions were presented and adopted, declaring the Massachusetts Medical Society in full accord with the American Medical Association, upon which Dr. Bronson, in behalf of that society, tendered its thanks for the unanimity with which the preamble and resolutions were carried.

The Committee on Ethics presented their report, in which, among other things, the names of certain members were mentioned for exclusion for non-payment of dues; and also the same action in regard to Dr. D. W. Bliss, who is under sentence of expulsion from the society of D. C.; also, that alumni associations of medical colleges were not entitled to be represented in the association. The subject of the contracting for the supplying of medical attention by the year to every one other than charitable institutions was laid over.

The question of the admission of females to positions as students and professors, in various medical institutions, was then referred to, and after a recital of the past action of the conventions on the subject, mention was made of the names of the Academy of Medicine, of Washington, D. C.; the Freedmen's Hospital, of Washington, D. C., and the medical department of the Howard Institution, as associations which had females among their practitioners, and had among their members unlicensed practitioners. The committee recommended that the delegations from these institutions be not, therefore, received in the present body.

Dr. Rayburn, of the District of Columbia, one of the professors of Howard University, took the floor against the recommendation of the committee. He alleged that if the delegates referred to were kept out, they would be kept out because they were not in good standing with the Washington Medical Association, and that that association licensed irregular practitioners, and also charged a fee for its license, against the legality of which there had been several judicial decisions.

The Howard University, he said, received all who applied for medical education, without distinction of color or of sex. If the association see fit that institutions of that class shall not be represented, of course they have the power so to act, but, at the same time, it should consider well what it was doing before taking such a step. He thought that every human being should be allowed the right to the very highest development that God has made him capable of.

Dr. Busey, of Washington, followed him in the defense of the medical society of that city, and in favor of the recommendation of the committee. He asserted that the society had never licensed irregular practitioners, or homœopathists, or any practitioner that had not presented a diploma from some regular medical school, or passed an examination before the society's board. Dr. Rayburn, on the contrary, was a member of an institution which did receive irregular practitioners.

Dr. Parmer, of Washington, from the Howard University, spoke on behalf of that institution. He denied that there was a lady member of the faculty, but that a lady had been employed as ophthalmologist. He acknowledged, however, that the lady spoken of was ophthalmic surgeon of an institution in which certain members of the faculty of Howard University are in daily consultation.

At the conclusion of this address, a great deal of confusion was occasioned by cries of "question," and by hisses from those who were anxious to vote without further discussion.

Dr. Parmer explained his position as professor in the Howard University. He said he had not practiced medicine in that city, and, therefore, had not procured a license, as the law requires. He did not understand why he should be excluded from this association on account of his connection with that institution.

A very rambling and animated discussion followed, and the President had great difficulty in preserving order. Motions were made in rapid succession, but they were not noticed by the chairman.

Dr. Sayre, of New York, moved that the resolution of the committee be adopted.

Dr. Todd seconded the motion, and, under the call of the previous question, it was finally carried by an overwhelming majority, whereupon the convention adjourned.

In the afternoon the various sections met as on the two previous days, for the consideration of medical and scientific subjects.

The members subsequently repaired to the University of Pennsylvania to partake of an entertainment tendered them by the professors and graduates of that institution.

In the evening, a lecture on Sound was given by Dr. F. Solis Cohen, at the Jefferson Medical College. The doctor illustrated his remarks by the use of various ingenious and delicate apparatus, showing, in one case, the waves of sound through the intervention of light thrown upon a mirror. The lecture room was crowded with the delegates and invited guests.

After the lecture, a handsome entertainment was given to the members of the convention and their ladies by Col. Thomas A. Scott at his residence.

FRIDAY.

The association convened in the same church and was called to order at the same hour by the President, Dr. Yandell, in the chair.

Resolutions of regret were offered for the death of Dr. Zeno Pitcher, of Detroit; Dr. W. W. Gerhard, Dr. Samuel H. Dickson, and Dr. Samuel Jackson, of Philadelphia.

Dr. Porter, of Delaware, spoke on the resolutions, paying a high tribute to the memory of Dr. Gerhard. The resolutions were unanimously adopted by a standing vote.

A resolution providing that the Marine Hospital Service be placed upon the same footing as the medical department of the U. S. Army, was laid over until the next meeting.

The reports of the various sections were presented, and without being read were referred to the Committee on Publication.

Reports were received from the following Special Committees, and referred, without reading, to the Committee on Publication:

On the Cultivation of the Cinchona Tree, Dr. L. J. Deal, of Philadelphia, chairman.

On the Structure of the White Blood Corpuscles, Dr. J. G. Richardson, Pennsylvania, chairman.

On Correspondence with State Medical Societies, Dr. N. S. Davis, Illinois, chairman.

Professor Gross offered an amendment to the by-laws, modifying the third section, so that instead of a report on Medical Education, or Medical Literature, or Climatology and Epidemic Diseases, there shall be annually delivered before the association, at its general meetings, an address on Medicine, one on Surgery, one on Midwifery, or the Diseases of Children, the lecturers to be appointed by the Committee on Nominations.

Laid upon the table till the next annual meeting.

Dr. Hartshorne offered a resolution, which, among other things, recommended the appointment of a commission of experts by the courts in all capital criminal cases where medical testimony is needed, instead of the present system of employing physicians as witnesses on opposite sides.

Adopted.

Dr. Askew, of Delaware, was appointed to prepare a series of resolutions expressing the feelings of the association upon the death of Prof. Samuel Jackson.

Resolutions of thanks were passed to the officers of public institutions, of the Naval Station, and to the authorities and congregation of the First Reformed Presbyterian Church Association. Also, to the Committee of Arrangements, medical profession, faculties and officers of the colleges, Col. Thomas A. Scott, Dr. Wm. Pancoast and Dr. Hugh L. Hodge, and to the retiring President, Dr. D. W. Yandell.

Dr. John Morris, of Baltimore, also moved that a vote of thanks be returned to the members of the press of Philadelphia, for their faithful attendance during the sessions.

Adopted.

A resolution was introduced setting forth that the association acknowledges the right of women to study and practice medicine and surgery in all the branches, but condemns the public association of the sexes in mixed classes and clinics as subversive of true modesty and delicacy in both sexes.

The consideration of the resolution was indefinitely postponed.

The formal business of the association being gone through with, the Chair addressed the convention in words of thanks for their support of him and his decisions during the sessions of the association. He closed by hoping to meet all the members of the association again on the other side of the Mississippi, at the next annual gathering. He then declared the Convention adjourned *sine die*.

By the many trains which left the Reading Railroad Depot during the afternoon, hundreds of the members of the Convention visited the Park, and amused and entertained themselves by driving to various parts of that section of the city, in lounging beneath the trees of the Belmont Mansion, and in partaking of temporary refreshments, preliminary to a collation which had been prepared for them in the pavilion.

This part of the ceremonies of the day came off at 4 o'clock, and the delegates, their ladies and invited guests, passed a most delightful hour in the pleasurable business of partaking of the luxuries of the season—a fitting termination to a professional business reunion.

At the close of the entertainment, addresses were made by John C. Cresson and John Welsh, Esqs.

